# Off-Label Cyproheptadine in Children and Adolescents: Psychiatric Comorbidities, Interacting Variables, Safety, and Risks of Hepatotoxicity

**DOI:** 10.7759/cureus.33745

**Published:** 2023-01-13

**Authors:** Mayank Gupta, Nihit Gupta, Jayakrishna Madabushi

**Affiliations:** 1 Child and Adolescent Psychiatry, Southwood Psychiatric Hospital, Pittsburgh, USA; 2 Psychiatry, Dayton Children's Hospital, Dayton, USA; 3 Psychiatry, Alabama Psychiatry, Birmingham, USA

**Keywords:** cyproheptadine, periactin, migraine prophylaxis, child and adolescent psychiatry, mental health illness, hepatic toxicity, appetite stimulation, migraine disorder

## Abstract

Cyproheptadine is a widely prescribed first-generation antihistamine, and due to its unique chemical structure, it has a popular off-label choice for a range of clinical conditions. Its efficacy is widely debated in the literature, but there are reports of hepatotoxicity as a rare adverse effect. Its benefits in migraine prophylaxis and appetite stimulation also underscore a highly overlapping clinical pathway that requires additional assessment and evaluation. The evidence suggests a baseline metabolic profile before initiation and assessment for co-occurring mental health conditions may improve overall response with evidence-based mental health interventions.

## Introduction

Cyproheptadine, a pharmacological agent from the piperidine family and a subgroup of first-generation antihistamines, has been widely used since the 1960s. Cyproheptadine is a potent, competitive, antagonist, H1 histaminergic receptor antagonist, an M1 muscarinic receptor antagonist, 5HT2A serotonergic receptor antagonist, and reportedly a calcium channel blocker [1].

Its approved clinical use is for allergic rhinitis but interestingly it is widely prescribed off-label to adults, children, and adolescents for many other clinical conditions. The unique effects are attributed to the chemical structure of tricyclic benzocycloheptene, i.e., cycloheptenes, with additional benzene rings. Its chemical structure closely resembles that of pizotifen, ketotifen, and tricyclic antidepressants (TCA). Cyproheptadine's two most noteworthy off-label uses are for appetite stimulation and migraine prophylaxis due to its orexigenic and anti-serotonergic properties, respectively [2]. There are many other uncommon off-label uses of cyproheptadine. For example, cyclical vomiting syndrome in infants, akathisia, tardive dyskinesia in adults, and antidote for serotonin syndrome [3,4]. Due to its sedative antihistaminergic effects, it is often prescribed for insomnia. Cyproheptadine is also studied for stimulant-related appetite suppression and insomnia in children and adolescents with attention-deficit/hyperactivity disorder (ADHD) [5]. This “off-label use" refers to the use of drugs for unapproved indications or age groups. Pediatric drug development is a complex process involving many stakeholders and restrictive regulatory challenges. A low or insufficient level of evidence is usually the basis for off-label use. In general, off-label use is common and legal [6], unless it violates ethical guidelines or safety regulations, in which case there could be legal liabilities and potential health risks. About 47% of psychopharmacological agents used in the children and adolescent population are off-label [7].

Although the off-label use of cyproheptadine has limited empirical evidence, its affinity for other receptors is the likely reason for undesired adverse effects. The merits of the knowledge about psychiatric comorbidities with neurological conditions like migraines are not discussed in the context of cyproheptadine, given the complex association with various interacting variables. In the last few years, there are empirical literature linking migraines with co-occurring mental health conditions [8]. These conditions include many but are not limited to autism spectrum disorders [9], ADHD [10,11], affective illness [12], anxiety, and eating disorders. In real-world clinical settings, chronic persistent headaches and or primary headaches often present with a mixed range of mental health symptomatology [13] leading to diagnostic challenges and treatment conundrums. Therefore, it is imperative to make inquiries into these complex relationships, risks, and benefits of off-label cyproheptadine use in children and adolescents with co-occurring mental health conditions. Cyproheptadine has an affinity for a wide range of receptors [14-22] and many proposed mechanisms of action to support its off-label use [23-28].

## Case presentation

A 14-year-old male was referred by his pediatrician to the outpatient child and adolescent mental health services for worsening symptoms of anxiety, school refusal, and marked inattention, which were attributed to his low school grades. During the psychiatric assessment, he reported that the symptoms first started two years ago but there were no prior treatments due to a lack of disclosure. The symptoms got worse after the school sent a truancy notice, prompting the family to seek help. During the review of systems, he was positive for loss of appetite and chronic underweight with a BMI of 17.1. He also reported bullying victimization and being called names since he was underweight. He lived with a family of working-class parents, and a 10-year-old younger brother in a rural community. He had a history of chronic persistent headaches and was also diagnosed with migraines by his primary care physician. Cyproheptadine 8 mg was prescribed by the pediatrician for appetite stimulation and migraine prophylaxis for the last two years.

On initial lab workup, aspartate aminotransferase (AST, 70 U/L) and alanine aminotransferase (ALT, 85 U/L) were elevated, which led to further inquiries and consultation with his primary care physician. Subsequently, his family physician initiated a workup including an ultrasound but there were no identifiable abnormalities. After a literature review on cyproheptadine, its hepatotoxic effects were discussed with the family, and it was tapered off.

In a short span of two weeks, his AST and ALT returned to the baseline. He was also started on sertraline 50 mg for anxiety. In addition, he also had weekly sessions of cognitive behavioral therapy and dietary consultation. The objective measurement of ADHD symptoms in two settings did not meet the criterion for a formal diagnosis but was monitored closely. In addition, interventions placed by the school counselor to address bullying helped with attendance, and a 504 plan with reasonable accommodations (Section 504 of the Rehabilitation Act of 1973) was initiated. At the 12-week follow-up, there was a 60% reduction in the symptoms of anxiety (measured on the Screen for Child Anxiety Related Disorders (SCARED)), weight gain of 7 lbs, and no reports of any symptoms of headaches.

## Discussion

The primary objective is to understand the nature of the association between primary headaches like migraines with mental disorders. Secondly, the study explores the following question: Is the assessment and treatment of co-occurring mental health conditions sufficient without needing headache prophylaxis? Lastly, the objective is to understand, amidst newer scientific developments, the potential risk of adverse effects and benefits of ongoing cyproheptadine off-label use including appetite stimulation. Table 1 summarizes the off-label uses of cyproheptadine.

**Table 1:** Off-label uses and the proposed mechanism of action 5HT2A: a subtype of serotonin (5-hydroxytryptophan) receptor; M1: a type of muscarinic acetylcholine receptor; ADHD: attention-deficit/hyperactivity disorder; IBS: irritable bowel syndrome.

Indication	Evidence based on recent studies
Migraine [14-16]	The data concerning cyproheptadine for prophylaxis was insufficient. The simultaneous inhibition of both serotonin 1B and 1D receptors and prevention of neurogenic inflammation caused by stimulation of the trigeminal nerve in patients with refractory migraine.
Appetite stimulation [17,18]	A safe, generally well-tolerated medication that facilitates weight gain in patients from a variety of underweight populations. Antagonism of serotonin in the appetite center of the hypothalamus may account for cyproheptadine's ability to stimulate the appetite.
Cyclical vomiting syndrome [19]	In 2008, the North American Society for Pediatric Gastroenterology, Hepatology, and Nutrition Consensus Statement recommended cyproheptadine for children < 5 years of age.
Akathisia [20,21], tardive dyskinesia [22]	5HT2A and M1 receptor blockade are known for cyproheptadine use for antipsychotic-induced akathisia.
Serotonin syndrome [23,24]	5HT2A antagonistic actions have been used as an antidote for cases with serotonin syndrome.
Insomnia [25]	Cyproheptadine does not have any considerable preventive effect on sleeping and appetite disorders induced by methylphenidate in ADHD children.
Functional gastrointestinal disorders (FGIDs) [26]	It effectively improves symptoms of functional abdominal pain, dyspepsia, abdominal migraine, IBS, and cyclic vomiting syndrome.
Anorexia nervosa [27,28]	Weight gain in restrictive anorexia nervosa and avoidant/restrictive food intake disorder (ARFID).

A growing body of scientific literature has linked migraine [29] with major depressive disorder, bipolar disorder [30,31], panic disorder [32], and social phobia generalized anxiety disorder [33]. Besides shared genetic pathways [34], bidirectional relationships between affective illness, anxiety disorders, and primary headaches like migraines and tension-type headaches are reported [35,36]. Therefore, the detailed mental health assessment of other co-occurring conditions may yield necessary information on the etiology of these clinical symptoms. Migraine is also known to be more prevalent in individuals with anorexia nervosa (AN) and bulimia nervosa (BN) [37,38]. The possibility that migraine may constitute an independent risk factor for the occurrence of eating disorders in young females has also been investigated [39,40]. Since studies have associated migraines and headaches with eating disorders, anxiety, and affective psychopathology, further research is needed to understand these relationships [41,42]. See Table 2 for details about these studies.

**Table 2:** Studies with the association between migraine and psychiatric disorders

Studies/authors	Summary of evidence linking migraine with psychiatric conditions
Radat et al. (2005) [29]	Migraine’s association was strongest for major depression and anxiety disorders (particularly panic and phobia), and comorbidity has also been reported with substance abuse and certain mood disorders.
Ortiz et al. (2010) [30]	Migraine is prevalent among individuals with bipolar disorder, particularly among bipolar disorder II. It is associated with an increased risk of suicidal behavior and comorbid anxiety disorders.
Zarcone et al. (2017) [31]	In the study, authors found that individuals with both migraine and epilepsy have a higher prevalence and incidence of anxiety, depression, and suicidal ideation.
Lampl et al. (2016) [33]	The authors concluded that depression and especially anxiety are comorbid more than by chance with migraine.
Dresler et al. (2019) [36]	Migraine’s associations with psychiatric comorbidities are complex, with a bidirectional association between major depression and panic disorder.
Giri et al. (2022) [41]	There is a bidirectional relationship between anxiety, depression, migraine, and tension-type headaches. The bidirectional association with anxiety was slightly stronger for migraine than tension-type headaches.

Cyproheptadine's use for migraine prophylaxis was based on its close resemblance with TCA and modulation of the serotonergic effects. Its ongoing use of migraine prophylaxis without the support of empirical studies is often widely debated and critiqued [43]. An influential study highlighted that many patients lack self-awareness about their diagnosis of migraine. There are fewer than expected clinicians who are making accurate diagnoses including specialists and misdiagnosis is common [44]. The overuse of diagnostics tests, unwarranted neuroimaging, and ambiguity about therapeutics are reported [45,46]. Given the strong and growing evidence of these associations, the children and adolescents diagnosed with migraine may benefit from additional assessment of the burden and psychosocial impairment related to psychiatric comorbidities. In our case, after mental health conditions were adequately treated and psychosocial stressors were mitigated, subsequently there was no further need for migraine prophylaxis.

There are many commonly reported adverse effects of cyproheptadine, including anticholinergic effects in young children, which could influence the quality of life. The orexigenic effects are not without a few serious adverse effects, including hepatoxicity [17]. In our case, the elevation in the liver transaminases was noted as an incidental finding during the initial workup, which resolved two weeks after cyproheptadine discontinuation. However, the idiosyncratic hepatoxic potential of cyproheptadine (possibly due to its effects on oxidative phosphorylation) is classified as category C in LiverTox [47]. In animal studies, an increase in the hepatic microsomal cytochrome P450 levels and structural changes in the liver cells are suggestive of its molecular level hepatotoxic pathways [48]. According to a systematic review, hepatic complication was reported in 1.4 out of 1,000 patients [47].

Therefore, patient selection remains critical before using cyproheptadine, and given its hepatotoxic effects, a baseline metabolic profile is recommended, with subsequent monitoring during treatment [47]. The evidence-based treatment of co-occurring mental health conditions and dietary modifications could be beneficial. Also, trials with newer, tolerable, and favorable adverse effects profiles should be the first-line therapeutics for migraine and weight gain [49]. The 2019 American Academy of Neurology (AAN) and the American Headache Society (AHS) practice parameters for pediatric migraine prevention recommend children and adolescents with migraine should be screened for mood and anxiety disorders because of the increased risk of headache persistence [49]. Cyproheptadine use is not recommended by the AAN and AHS practice guidelines for migraine prevention. Likewise, the treatment for children with failure to thrive (FTT) may require a comprehensive evaluation, and cyproheptadine is recommended only for specific populations with cystic fibrosis, chronic renal disease, or patients undergoing treatment for malignancy [50]. Appetite stimulants are not recommended for most children with FTT [50].

## Conclusions

The therapeutic potential of cyproheptadine has been established based on its decades of use for a myriad range of disorders and is perceived as a safe option. It is frequently prescribed for clinical conditions like appetite stimulation and migraine prophylaxis and is frequently linked with co-occurring mental disorders. There is no consensus on the clinical profiles of those likely to benefit from cyproheptadine use; similarly, there are no studies that examine its long-term efficacy and risks. The mere treatment of symptoms, without a detailed assessment of other conditions and contextual understandings, has potential pitfalls. Both common and serious adverse effects are associated with cyproheptadine, and monitoring the metabolic profile remains critical even when the benefits outweigh the risks. The cyproheptadine use may be time-limited, but chronically persistent symptoms of loss in appetite and headaches could be a signal needing further attention. The limitless therapeutic potential of this drug and its overlapping pathways inspire scientific inquiries to test these plausible hypotheses.
